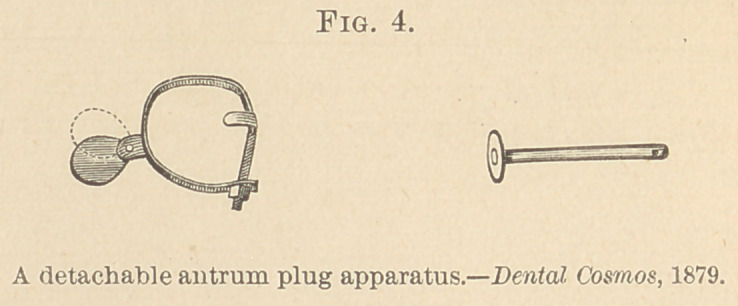# Anæsthetic Operation for the Treatment of Diseases of the Antral Chamber

**Published:** 1891-07

**Authors:** J. N. Farrar

**Affiliations:** New York City


### International Dental Journal.

Vol. XII. July, 1891. No. 7.

### Original Communications.[1]

### ANÆSTHETIC OPERATION FOR THE TREATMENT OF DISEASES OF THE ANTRAL CHAMBER.

BY J. N. FARRAR, M.D., D.D.S., NEW YORK CITY.

In the *Dental Cosmos* for March, 1884, is published a paper explaining my plan of treatment for the cure of some cases of alveolar abscess by amputation of more or less of the abscessed root of the tooth, and several original cases are illustrated by section, to show the appearance of the tooth immediately after the operation. (See Fig. 1, from the paper.)

In the present article my object is to publicly answer inquiries concerning the operation upon this plan for reaching and treating the antral chamber. The plan, which dentists sometimes complimentarily call by my name, consists in amputating the "abscessed root" that caused the antral trouble, leaving the remainder of the tooth undisturbed;[2] and this plan, of course, shows its best results in cases where the offending tooth has more than one root.

I regard this operation as superior to that of extracting the

---

[1] The editor and publishers are not responsible for the views of authors of papers published in this department, nor for any claim to novelty, or otherwise, that may be made by them. No papers will be received for this department that have appeared in any other journal published in the country.

[2] In vol. vi. (1881) of the Proceedings of the Medical Society of the County of Kings (Brooklyn, New York) is a similar paper by me on the treatment of antral disease.

entire tooth, as illustrated by Fig. 2,[1] because by amputating the offending root the operation as fully rids the case of the cause of the antral trouble as would the extraction of the entire tooth, and it furnishes equally free passage for the nozzle of the spray-syringe to the antral chamber.

Fig. 3 illustrates a case showing the plan of the operation. A represents the right antral chamber; T, the tooth that caused the disease; C, the locality of the abscess; R, the place left after amputating the palatine root, which caused the trouble; S, the nozzle of the syringe spraying the chamber; O, floor of the orbit of the right eye.

Of course a tooth that has lost one of its roots cannot be quite as firm as when all of it was healthy and normal, but several years of experience show that such teeth are not only valuable, æsthetically, but are by a little care useful for mastication purposes.

The first conception of this operation was suggested in the case of a public speaker, to whose personal appearance the loss of a tooth would have been great injury. By amputating the offending root, I found that the passage-way to the antrum was at once opened, and the chamber were easily reached with the spray-syringe. The medicines used in this case was chloride of sodium dissolved in water, and chloride of zinc (solution).

### ADJUSTABLE METALLIC PLUGS FOR PASSAGE-WAYS TO THE ANTRAL CHAMBER WHILE UNDER TREATMENT.

In the *Dental Cosmos*, vol. xxi. (1879), page 556, begins a paper on the construction and application of several forms of detachable antral plugs, made of metal, to prevent premature closing of passages from the oral cavity to the antral chamber during the treatment.

Fig. 4 illustrates one of these devices.

A later modification of the plugging apparatus, devised by me for a case in which the offending root was amputated, consisted of a piece of smooth round platinum wire, slightly larger than the tube of the syringe, and oval at the upper end. This plug, like some of the older ones referred to, was held in place by a clamp-band around the crown of a tooth (in this case the offending tooth). The connection of the plug with the clamp-band was made

---

[1] Taken from one of my early papers, published in the *Missouri Dental Journal*, 1879. This also illustrates the first spraying instrument for the antral chamber.

by a short piece of pliable platinum wire of much smaller size. These three parts were soldered together in such a relation that the plug rested easily in the canal, and caused no injurious lateral pressure upon the tissues constituting its walls. Before syringing the antrum the plug was removed, and it was returned after the operation.

## Figures and Tables

**Fig. 1. f1:**
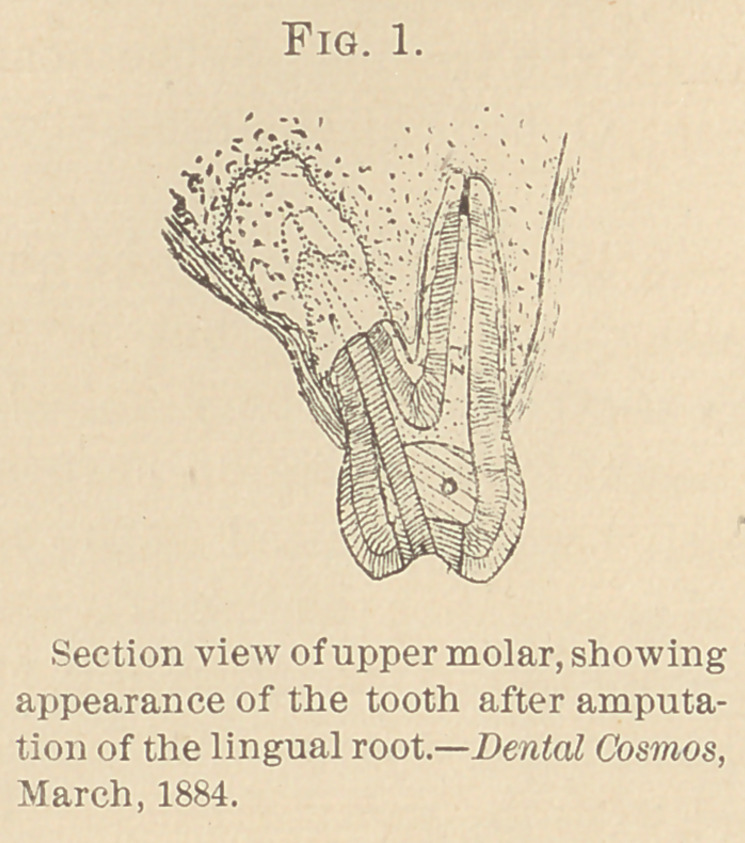


**Fig. 2. f2:**
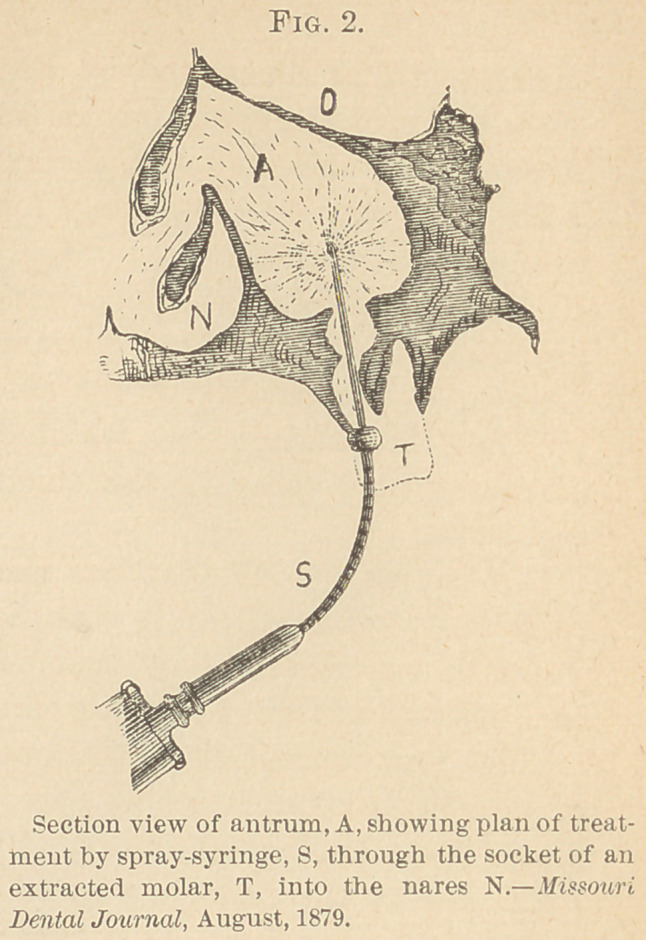


**Fig. 3. f3:**
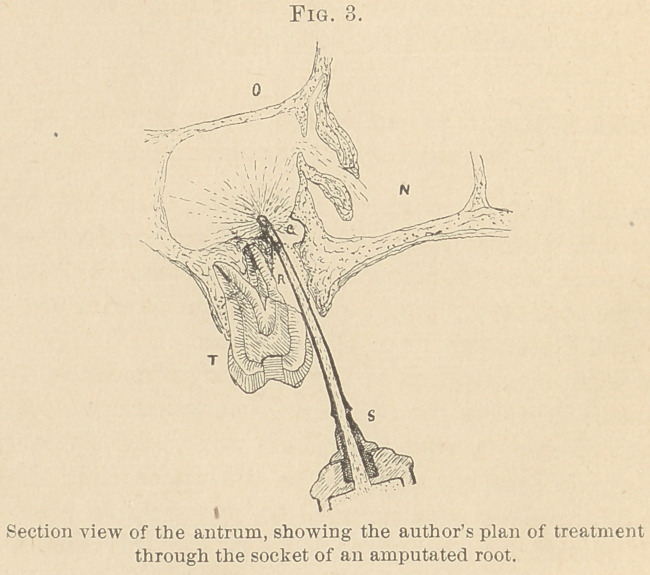


**Fig. 4. f4:**